# Impact of the manufacturing process on the commutability of erythropoietin control materials for external quality assessment schemes

**DOI:** 10.3389/fmolb.2026.1773497

**Published:** 2026-04-08

**Authors:** Luisa Toll, Patricia Kaiser, Ingo Schellenberg, Mario Thevis, Folker Wenzel

**Affiliations:** 1 INSTAND e.V., Society for Promoting Quality Assurance in Medical Laboratories, Düsseldorf, Germany; 2 Institute of Biochemistry/ Center for Preventive Doping Research, German Sport University Cologne, Cologne, Germany; 3 Institute of Bioanalytical Sciences (IBAS), Center of Life Sciences, Anhalt University of Applied Sciences, Bernburg, Germany; 4 Faculty II: Engineering and Technology, Furtwangen University, Villingen-Schwenningen, Germany

**Keywords:** commutability, control material, erythropoietin (EPO), external quality assessment (EQA), immunoassays

## Abstract

**Introduction:**

Erythropoietin (EPO) is a measurand that has not yet been sufficiently standardized in laboratory medicine. This is why quality control within the framework of external quality assessment (EQA) schemes is of great importance. Therefore, the INSTAND e.V. EPO EQA was introduced to support quality assurance of medical laboratories for this measurand. The use of commutable control material (CM) enables the comparison of various measurement systems with one another and allows to assess the level of harmonization between laboratory measurements. This study assessed the suitability of different EPO CMs for use in EPO EQA schemes and the impact of sample preparation, such as dilution, spiking and lyophilization.

**Methods:**

Eleven candidate CMs in both frozen and lyophilized forms were analyzed and compared to 80 clinical samples (CS) using a chemiluminescence immunoassay (CLIA) and two different enzyme-linked immunosorbent assay (ELISA) test kits. The commercially available CMs were based on a serum pool that was either diluted with human serum albumin or enriched with various recombinant EPO (rhEPO) preparations. Commutability was assessed oriented towards the principles of difference in bias analysis based on the recommendations of the International Federation of Clinical Chemistry and Laboratory Medicine (IFCC) Working Group on Commutability.

**Results:**

Most CMs remained within the range of the CS bias distribution across all paired method correlations. However, CMs exceeding the limits were also observed in some cases, even the native frozen pooled serum. Dilution and lyophilization affected the inter-method bias to varying degrees. Bias values for CMs enriched with different rhEPO preparations were mostly within the same range, though for CMs containing darbepoetin alfa in some cases higher inter-method bias was observed. Apparent differences in non-selectivity between the assays limited the ability to reliably assess commutability.

**Conclusion:**

The findings highlight current methodological challenges regarding the manufacturing of suitable materials and commutability assessment. Further investigations are needed regarding the standardization and harmonization of EPO measurements. Until further data is available, the evaluation within the EPO EQA should remain within method-specific sub-collectives.

## 1 Introduction

Erythropoietin (EPO) is a glycoprotein hormone that regulates the production of erythrocytes by stimulating their proliferation, differentiation and survival. It is primarily synthesized in the kidneys and is essential for maintaining oxygen homeostasis. EPO measurement can be used clinically to assess anemia, especially in patients with chronic kidney disease, where decreased endogenous production is a major contributing factor to the development of anemia (Bunn, 2013; Portolés et al., 2021). Furthermore EPO testing provides diagnostic insight into erythrocytosis whereby suppressed EPO concentrations are typically observed in polycythemia vera and elevated levels are characteristic of secondary erythrocytosis (McMULLIN, 2008). Clinical laboratories commonly use a range of immunoassay systems to quantify serum EPO. However, measurement results have been shown to vary significantly depending on the laboratory and the method used (Toll et al., 2024). Calibrators for commercial assays can be traceable to one or more different available international standards, which consist of recombinant erythropoietin (rhEPO) synthesized in various cell lines. Differences in the cell lines used for rhEPO production may contribute to discrepancies between methods (An et al., 2019). This underlines the need for harmonization and standardization.

One of the primary objectives of laboratory medicine is to ensure patient safety and to support accurate clinical decision-making. DIN EN ISO 15189:2022 stipulates that clinical laboratories are required to monitor the performance of their measurement procedures through interlaboratory comparison, typically achieved by participating in external quality assessment (EQA) schemes (DIN EN ISO 15189, 2022). Samples of undisclosed analyte concentrations are distributed to the participating laboratories as part of these EQA schemes. The measurement results are submitted to and analyzed by the EQA provider, who offers feedback on the performance of the individual laboratories (Montalvão et al., 2022). EQA providers themselves must be accredited according to ISO/IEC 17043:2023, which sets forth the international requirements regarding competence, impartiality, and quality management (DIN EN ISO 17043, 2023).

The use of true clinical samples in EQAs is often limited by insufficient volumes or the need for specific analyte concentrations. For this reason, pooled or enriched samples are often used as alternative materials. Additionally, stabilizing techniques like lyophilization or the use of stabilizing additives is commonly used to improve storage stability of protein-based materials (Emami et al., 2018). However, these artificial samples do not necessarily mimic the characteristics of true clinical samples (Laudus et al., 2022). The use of commutable control samples is essential to ensure a meaningful assessment of laboratory performance and to limit material-dependent effects on the measurements. In contrast, non-commutability limits the evaluation of EQA results by complicating the interpretation of results (Miller, 2003).

According to the International Vocabulary of Metrology, commutability is a property of a reference material that indicates agreement between the measurement results for that material obtained using two specified measurement methods, and the measurement results for other specified materials–typically true clinical samples (JCGM, 2012). To assess commutability, several approaches have been developed that generally focus on the statistical agreement of these sample types to a panel of clinical specimens across different measurement systems. One approach recommended by the International Federation of Clinical Chemistry and Laboratory Medicine (IFCC) Working Group on Commutability relies on paired measurements of both clinical samples and candidate EQA materials using at least two different measurement procedures. Based on this data, the bias of the EQA materials is calculated relative to the clinical samples and the differences in bias are evaluated. If these differences fall within predefined acceptability limits, the material is considered commutable (Miller et al., 2018; Nilsson et al., 2018). For control material (CM) used in EQAs, these limits should be derived from the statistical distribution of results from clinical samples, so that a commutable CM falls within the expected variability observed in real patient samples (Sandberg et al., 2023).

INSTAND e.V. introduced its EPO EQA scheme in 2017 and has conducted it on a biannual basis ever since. The immunological methods mainly used by the participating laboratories in this EQA are chemiluminescence immunoassay (CLIA) and enzyme-linked immunosorbent assay (ELISA). In this EPO EQA, the measurement results for the CMs are based on the deviation from consensus values within individual peer groups. This approach is suitable for monitoring the performance of individual analytical procedures. However, with the aim of patient safety, the optimal assessment of harmonization across EPO measurement procedures would ideally be based on an evaluation of the entire participant group. This requires commutable CMs (Sandberg et al., 2023). The aim of this work was to investigate the suitability of potential materials for this EPO EQA. In the context of this study, the impact of EPO CM preparation on the measurement results was evaluated. The commutability of these materials was assessed using ELISA and CLIA measurements and based on the general principles of the IFCC recommendations.

## 2 Methods

### 2.1 Sample preparation

The EPO CMs used in this study were commercially available samples provided by the manufacturer in.vent Diagnostica GmbH (Henningsdorf, Germany). According to the manufacturer, the CMs were derived from a human serum pool with physiological EPO concentrations. A low-level sample (<5 IU/L), prepared through dilution with human serum albumin (HSA) solution, as well as higher-level samples (>60 IU/L) were provided. These higher concentration samples were enriched with either epoetin alfa, epoetin beta, epoetin zeta or darbepoetin alfa. Each CM was supplied in frozen and lyophilized form, except for the HSA-diluted sample, which was only available as a frozen preparation due to clotting observed upon reconstitution. All samples were stored at −20 °C until analysis. Details on the different CMs, sample components and preparation can be found in Table 1.

**TABLE 1:** Classification of control materials (CM) 1–11.

Control material	Sample components	Preparation
CM1	PS	Lyophilized
CM2	PS	Frozen
CM3	PS diluted	Frozen
CM4	PS + epoetin α	Lyophilized
CM5	PS + epoetin α	Frozen
CM6	PS + epoetin β	Lyophilized
CM7	PS + epoetin β	Frozen
CM8	PS + epoetin ζ	Lyophilized
CM9	PS + epoetin ζ	Frozen
CM10	PS + darbepoetin α	Lyophilized
CM11	PS + darbepoetin α	Frozen

PS, pooled serum.

### 2.2 Erythropoietin measurements

The measurement systems used in the study were representative of those measurement methods mainly used in the EPO EQAs. These were a CLIA by Atellica® IM Analyzer, Siemens Healthineers, Erlangen, Germany (CLIA), and two different ELISA Kits: R&D Systems, Minneapolis, MN, United States (ELISA A) and Stemcell Technologies, Vancouver, BC, Canada (ELISA B). Each assay was performed in a single laboratory setting.

The EPO concentration was measured in triplicate for 80 different anonymized residual clinical serum samples (CS) using all three assays. The eleven different CMs underwent 40 measurement repetitions with each method performed in alternation with the measurements of the CS and in the same measurement series. Because of insufficient sample volume and 5 failed measurements, CM3 was only measured 35 times by CLIA. No ELISA B results were obtained for CM5 and CM9 because the upper detection limit was reached for these two samples. This was also the case for three of the CM7 repetitions which explains why there are 37 instead of 40 measured values for CM7.

### 2.3 Data analysis and commutability assessment

Microsoft Excel (Microsoft 365, Version 2508 [Build 19127.20222]) was used for primary data processing. Further data visualization and commutability calculations were performed using RStudio (Version 2025.09.0 + 387, Posit PBC, Boston, MA, United States).

The sample size for this study was determined using a simulation approach as described by Miller et al. (Miller et al., 2023). Preliminary method comparison was performed using data obtained from 27 clinical serum samples measured in triplicates by all three methods. These results were used to estimate the repeatability of methods and the variability caused by differences in non-selectivity.

These variances together with an assumption of a potential non-commutability bias of 10% and an initial commutability limit of 20% were considered in the simulation. The statistical power was defined as the probability of correctly classifying a CM as commutable. Based on these simulations, the final sample size of 80 CS measured in triplicates and 40 replicates of the CM achieved a statistical power of approximately 80% under the above specified conditions.

Commutability was assessed using difference in bias analysis, oriented towards the general principles recommended by the IFCC Working Group on Commutability (Miller et al., 2018; Nilsson et al., 2018).

To consider variability of the results in parts of the measuring range, the analysis was split into two predefined concentration ranges (1–20 IU/L and 7–120 IU/L). The data were ln-transformed before undergoing further evaluation. CLIA, ELISA A and ELISA B were correlated in pairs, and the bias of the CM and the mean CS were calculated. The difference in bias (B) was then calculated (ΔB = B_CM_–mean B_CS_). Standard uncertainties of the mean measurement results were derived as the standard deviation per sample divided by the square root of the number of replicates. Combined standard uncertainties of CM and CS were calculated as the square root of the sum of square uncertainties ( $u_{c}=\sqrt{{u_{CS}}^{2}+{u_{CM}}^{2}}$ ), and expanded uncertainties (U) were obtained by multiplying with a coverage factor of 1.9 (95% confidence). A CM was considered commutable if its difference in bias, including the expanded uncertainty (ΔB ± U), met the predefined commutability criterion. If ΔB ± U lay completely outside these defined limits, the CM was deemed non-commutable. If ΔB ± U partially overlapped with the limits, the result was regarded as inconclusive (Miller et al., 2018; Nilsson et al., 2018).

Unlike the IFCC recommendations, which propose a prior definition of fixed commutability limits, the commutability criterion in this study was derived empirically. Initial sample size calculations considered a commutability limit of ±20%, but the final assessment used acceptance limits defined by the 2.5th and 97.5th percentiles of the CS bias distribution. This range covers 95% of the CS bias values. This adjustment considers the generally high variability observed in the EPO measurements and adapts the IFCC difference-in-bias concept without strictly applying it. Consequently, the applied approach does not allow for a rigorous demonstration of commutability according to the IFCC-model but rather aims to evaluate whether the CMs behave within the natural variability observed for routine clinical samples.

The effects of the various sample properties on the measurement results were examined using the logarithmic pairwise correlated bias values between the three methods. These were summarized for all methods in box plots, and the different samples were compared.

## 3 Results

**FIGURE 1:**
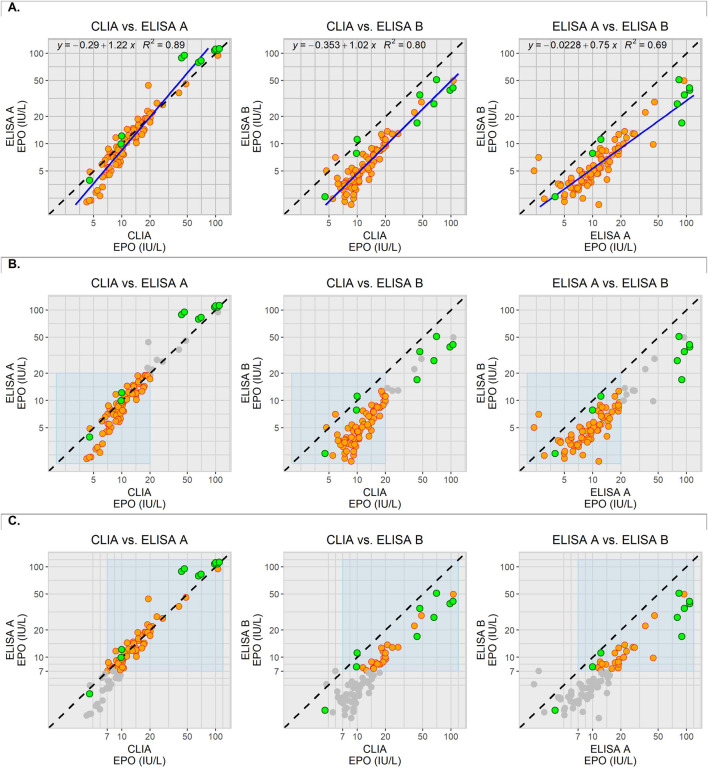
Pairwise correlation of the methods with the mean values of the measured EPO concentrations for the CS (orange) and CM (green). Error bars were omitted to provide visual clarity. The dotted black line represents the optimal agreement of the two correlated methods. The grey dots represent the CS data excluded for further commutability assessment. The x- and y-axes are displayed on a logarithmic scale. **(A)** Overall data distribution. The trend line (blue) refers to the data distribution of the CS. The linear equation and the coefficient of determination (*R* ^2^) refer to this trend line. **(B)** CS data (orange) for the lower concentration range (light blue area) of between 1 and 20 IU/L. Green dots falling out of the light blue area were not included in the evaluation of the respective concentration range. **(C)** CS data (orange) for the higher concentration range (light blue area) of between 7 and 120 IU/L. Green dots falling outside of the light blue area were not included in the evaluation of the respective concentration range.

Mean EPO concentrations of the CS ranged from a minimum of 4.2 IU/L for CLIA, 2.3 IU/L for ELISA A, and 1.1 IU/L for ELISA B to a maximum of 106.2 IU/L for CLIA, 94.5 IU/L for ELISA A, and 49.8 IU/L for ELISA B. Methods were correlated in pairs in order to assess the agreement or systematic differences between them (Figure 1A). Here, a dispersion of the measured values along the x-axis was visible.

In order to investigate commutability and to counteract disagreement between the methods, concentration ranges were defined within which separate evaluations were to be carried out (Figures 1B,C). The lower concentration range included all measurements between 1 and 20 IU/L. The measured values of CM1, 2 and 3 fell within this range. The upper concentration range was defined between 7 and 120 IU/L. This range included the measured values for CM1-2 and CM4-11. No measured values could not be recorded for CM 5 and 9 for ELISA B because the upper detection limit of the assay had been reached. Therefore, these could not be evaluated in correlation with ELISA B.

**FIGURE 2:**
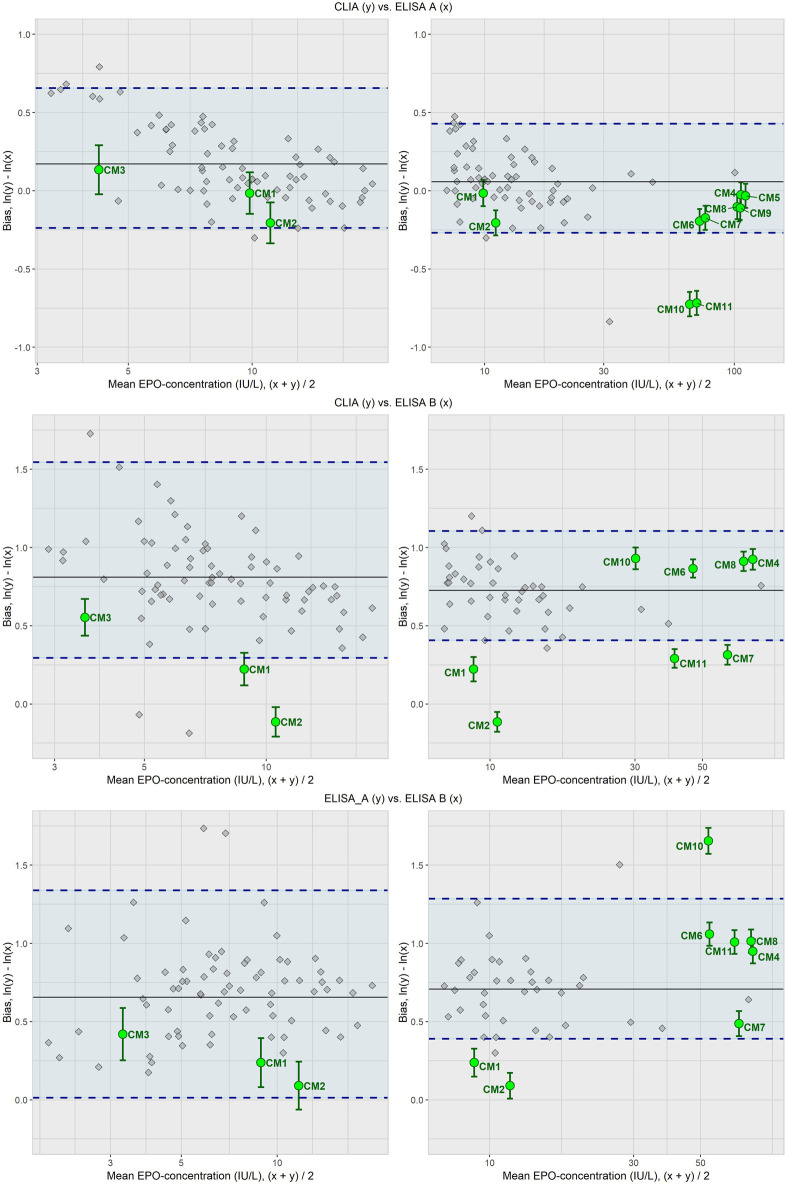
Difference in bias analysis of CM1-11. The grey squares show the bias between the respective methods correlated in pairs for each of the CS. The black line shows the mean bias between these methods for the CS. The blue dashed lines represent the commutability criteria set at the 2.5th and 97.5th percentiles of the CS bias distribution. The green dots are the mean bias between the two methods for the CMs, including bars that represent the uncertainty in the difference in bias between CM and CS mean bias. Classification of the CMs, including sample components and preparation, can be found in Table 1. No measured values could be recorded for CM5 and CM9 for ELISA B since the upper detection limit of the assay had been reached. Therefore, these could not be evaluated in correlation with ELISA B.

The main results for the bias comparison between the different CMs and the mean bias of the CS can be seen in Figure 2. In the lower concentration range, CM3 was within the defined limits across all method correlations. CM1 remained within the acceptance limits in most cases but for CLIA vs. ELISA B the mean difference in bias exceeded the limits, leading to an inconclusive outcome. CM2 (frozen serum pool) produced inconclusive findings in two comparisons and fully exceeded the limits in another (CLIA vs. ELISA B).

In the higher concentration range (7–120 IU/L), CM4, 6 and 8 consistently fell within the defined limits across all method correlations. In contrast, CM1 and CM10 only remained within the limits in one comparison each, while in all of the others they exceed the range of almost all the CS. CM2 again showed one inconclusive result but otherwise failed to meet the limits. CM11 only for the ELISA A vs. ELISA B correlation fell within the limits. CM7 complied with the limits in two comparisons but not in the third (CLIA vs. ELISA B). Both CM5 and CM9 remained within the range of the CS in the CLIA vs. ELISA A method correlation.

**FIGURE 3:**
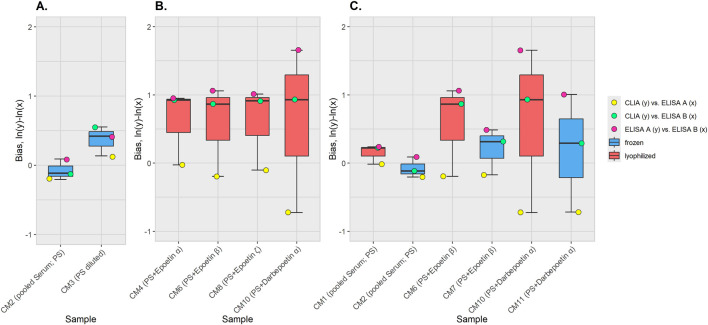
Bias between measurement methods for the different CM. Individual bias values are shown as points. Bias was calculated for: CLIA vs. ELISA A (yellow), CLIA vs. ELISA B (green), and ELISA A vs. ELISA B (pink). Boxplots summarize the distribution per sample and show the median (horizontal line), the interquartile range (IQR; box), and whiskers that extend to 1.5 times the IQR. The boxplots colors represent the sample condition: frozen (blue) or lyophilized (red). CM5 and CM9 were not included in the diagrams since no results were obtained for these two CMs with ELISA **(B)**. The classification of the CM, including sample composition and preparation, is provided in Table 1. **(A)** Bias comparison for the native pooled serum (CM1) and the corresponding diluted pooled serum (CM3). **(B)** Bias comparison for CM spiked with different rhEPO preparations. Only lyophilized CM are included here, as not all frozen CM produce results with ELISA **(B)** **(C)** Bias comparison between frozen and lyophilized CM.

The bias of the CM between methods was also assessed for the different sample types (Figure 3). Higher bias values were observed for the diluted sample (CM3) than for the base material (pooled serum, CM2) (Figure 3A).

Since no results were obtained for ELISA B for all frozen CM spiked with rhEPO, only the lyophilized CM were compared to assess the differences between the bias values due to spiking. These showed comparable bias between methods for the CM spiked with different epoetins (CM4, 6, 8). For the CM spiked with darbepoetin, a shift in values was observed in the method correlation CLIA vs. ELISA A and ELISA A vs. ELISA B (Figure 3B).

To asses the impact of lyophilization, the CM were compared pairwise (CM1 vs. CM2, CM6 vs. CM7, CM10 vs. CM11; Figure 3C) For all pairs, higher bias values were observed for the method correlations CLIA vs. ELISA B and ELISA A vs. ELISA B in the lyophilized compared to the frozen materials. Differences for the CLIA vs. ELISA A correlation were marginal.

## 4 Discussion

In the context of this study, not all of the CMs remained within the range of the CS’ bias distribution for every method correlation. This was for example the case for CM2. As the native frozen serum pool, CM2 was the least processed of all the materials. In contrast, far more processed CMs (lyophilized, diluted, enriched with rhEPO) are closer to the mean bias of the CS. This indicates that commutability cannot be universally inferred from processing level or material type.

The bias was consistently higher for the diluted CM (CM3) than for the native material (CM2). This may be due to lower EPO concentrations in CM3 than in CM2. It has been shown that bias values can be more distinct in lower concentration ranges for certain analytes (Koerbin et al., 2014). In contrast CM3 was nearest to the mean bias of the CS in the low concentration range. Indicating that dilution might not necessarily affect commutability. However, in order to make a definitive assessment of the influence of dilution, further samples would need to be tested at different dilution levels and in further concentration ranges. To date, data on commutability of EPO control materials remains limited. However, a study on a different protein investigating the impact of dilution of anti-β2-glycoprotein I IgG reference materials has reported no effect on commutability (Monogioudi et al., 2021).

Most CS enriched with rhEPO remained within the commutability limits across the different method correlations, except CM7, 10 and 11. Measured EPO concentrations for the different derivates did not always agree across all methods. This may be due to the structural heterogeneity and deviating molecular properties of the different rhEPOs compared to endogenous EPO, resulting in assay-dependent differences in antibody binding behavior. Previous studies have shown that cross-reactions and recovery of certain rhEPO variants vary between assays, indicating differences in selectivity towards the molecule variants (Mongia et al., 2007; Owen and Roberts, 2011). In particular, it was found that for CM10 and CM11, which were enriched with darbepoetin alfa, at least one of the two materials exceeded the bias range of the CS in all method correlations. For these two CM, ELISA A measured in a significantly higher range than ELISA B and CLIA. This led to a shift in bias values in method correlations including ELISA A. Darbepoetin alfa differs from endogenous EPO and Epoetin at five positions in its amino acid sequence, resulting in two additional binding sites for oligosaccharides. It therefore contains five N-linked carbohydrate chains (two more than endogenous EPO and Epoetin) leading to a higher molecular weight and sialic acid content as well as increased negative charge (Ibbotson and Goa, 2001; Egrie and Browne, 2002). The antibody binding immunological test systems might therefore also differ to a certain degree. In a study by Long et al., candidate EQA materials enriched with recombinant aminotransferases from varying sources showed differences in commutability, indicating that the origin of the recombinant material might be a relevant factor and should be taken into account (Long et al., 2021).

Lyophilization had a significant impact on bias between methods, albeit to differing extents. While lyophilization did not noticeably affect the bias between CLIA and ELISA A, and the frozen CMs remained close to their lyophilized equivalents, clear differences were observed in the other method correlations after lyophilization. Lyophilization may lead to changes in conformity and structure, as well as loss of protein activity and stability. Freeze-thaw and drying cycles within this process can cause denaturation, aggregation and surface adsorption of the relevant proteins. Additionally, chemical degradation processes such as oxidation, hydrolysis and deamidation can further compromise protein stability (Butreddy et al., 2021). However, it has been found that protein quality and quantity can be maintained even when lyophilized samples are stored for up to 20 months at 4 °C, and lyophilization can have a positive effect on reproducibility of measured concentrations (Molnar et al., 2021). Despite these differences, the majority of lyophilized CMs remained within the bias range observed for the CS.

Results indicate that EPO measurement range is highly dependent on method. While the results for CLIA and ELISA A had similar value ranges, ELISA B measurements were consistently lower, especially for CMs enriched with rhEPO. Variability in EPO measurements between methods and laboratories has already been demonstrated in a summary evaluation of past EQA results (Toll et al., 2024).

The observed deviation from the trend line, as well as the concentration-dependent divergence of values between the methods, suggests relevant differences in assay selectivity (Figure 1). Reliably demonstrating the commutability of CM becomes challenging when substantial differences in non-selectivity (DINS) are present. Insufficient selectivity and limited analytical specificity remain common challenges in routine clinical laboratory tests, especially in ligand-binding assays (Vogeser and Habler, 2024). According to Sandberg et al., excessive DINS between measurement procedures may restrict a stringent commutability assessment. Using the difference in bias approach is not possible in this case (Sandberg et al., 2023). Therefore, this work aims to evaluate the CM relative to the variability of CS, taking into account that, due to methodological heterogeneity, no definite conclusions can be made regarding commutability in this study. Considering possible strong DINS, the applied criterion was statistically derived based on the distribution of the CS measurements. The objective of this analysis was therefore to describe whether the CMs lie within the natural variability of real clinical samples rather than to formally determine commutability. Stricter limits might therefore not be applicable, especially due to the observed methodological limitations.

When determining EPO, a certain degree of variability in measurement results is not clinically relevant. According to the KDIGO Clinical Practice Guideline for Anemia in Chronic Kidney Disease, EPO levels are not routinely considered when differentiating EPO deficiency from other etiologies of anemia in patients with kidney disease. Instead, clinicians rather determine the total reticulocyte count when assessing erythropoietic proliferation activity (KDIGO, 2012). The clinical value of EPO in the context of polycythemia vera is also restricted since JAK2 mutation analysis provides a more specific diagnostic tool (McMullin et al., 2019). Accordingly, EPO is rarely considered in isolation in everyday clinical practice and is always viewed in conjunction with other biomarkers, such as hemoglobin. Furthermore, the current level of harmonization among EPO measurement procedures is still restricted (Toll et al., 2024). So, the requirements for an EPO CM may be less stringent than those of higher-order reference materials within metrological traceability chains or a CM for highly critical measurands where even minimal deviations have major consequences for patient care. Reference materials used as calibrators in traceability chains need stricter commutability limits, while wider limits may be set for CMs (Braga and Panteghini, 2019).

Furthermore, commutability is not a property that can be assumed as a matter of principle, even if it might apply for some materials of the measurand in question. Commutability findings can only be interpreted in the context of the study design. It is also highly dependent on the mathematical model chosen (Miller et al., 2018). Since commutability cannot be assumed for all materials of this measurand, commutability would need to be continuously reassessed for all newly introduced CMs. These commutability studies are based on theoretical statistical models that may be challenging to implement in routine practice. Such study designs are resource intensive, which may limit the feasibility of continuous implementation for every newly introduced CM (Vierbaum et al., 2024).

Besides the previously described notable DINS between the measurement procedures, further limitations of the study design need to be considered. As mentioned by Miller et al., the commutability findings are assumed to only be valid in the context of the study and only for the methods and conditions examined (Miller et al., 2018). Consequently, a restriction of this study is that only a limited number of detection methods and assay manufacturers could be examined. The measurement systems used in the study were selected based on prior experience indicating analytical reliability and on their availability at the time of the study. The selected assays aim to represent methods commonly used in routine EPO measurements. Inclusion of additional widely used immunoassays would be necessary to enable a more extensive assessment of commutability across different measurement procedures. Furthermore, no reference measurement procedure from the highest metrological order could be used to compare methods. Therefore, it was not possible to determine target values for the samples used that would be as close as possible to the true sample concentrations. No reference method is currently available for EPO. Since we had to rely on residual serum samples from a routine laboratory for the CS, it should also be mentioned that certain concentration ranges could only be included to a limited degree. The EPO measurements for the CS were mostly within the physiological range. However, there was a particularly limited number of CSs in the elevated concentration range. CM4-11 are mostly located within the higher concentration range. The limited representation of CS within this range further compromises commutability assessment. However, due to limited availability, it can generally be challenging to cover the entire concentration range with real patient samples.

## 5 Conclusion

This work highlights the challenges associated with commutability studies and shows that rigorous proof is not always possible. Strong DINS compromises both the production of commutable CMs, and the ability to monitor and improve agreement between different assays. This study addresses a measurand for which standardization and harmonization efforts remain limited. Despite these limitations, the results of this study provide valuable insights into the current challenges and highlight the need for further investigations. The study demonstrates that the consensus value must be used for evaluation on the basis of method collectives as long as no material is available for external quality control that is suitable for all EPO analysis systems currently on the market.

## Data Availability

The raw data supporting the conclusions of this article will be made available by the authors, without undue reservation.
